# Mitochondrial respiratory chain function and content are preserved in the skeletal muscle of active very old men and women

**DOI:** 10.1016/j.exger.2018.09.020

**Published:** 2018-11

**Authors:** R.M. Dodds, K. Davies, A. Granic, K.G. Hollingsworth, C. Warren, G. Gorman, D.M. Turnbull, A.A. Sayer

**Affiliations:** a AGE Research Group, Institute of Neuroscience, Newcastle University, Newcastle upon Tyne, UK; b NIHR Newcastle Biomedical Research Centre, Newcastle University and Newcastle upon Tyne Hospitals NHS Foundation Trust, Newcastle upon Tyne, UK; c Academic Geriatric Medicine, Faculty of Medicine, University of Southampton, Southampton, UK; d Newcastle University Institute for Ageing, Newcastle upon Tyne, UK; e Newcastle Magnetic Resonance Centre, Institute of Cellular Medicine, Newcastle University, Newcastle-upon-Tyne, UK; f Newcastle University Centre for Ageing and Vitality, Newcastle upon Tyne, UK; g Wellcome Trust Centre for Mitochondrial Research, Newcastle upon Tyne, UK

**Keywords:** Mitochondrial function, Sarcopenia, Physical performance, Muscle mass, Ageing

## Abstract

**Introduction:**

The loss of mitochondrial function and content have been implicated in sarcopenia although they have been little studied in the very old, the group in which sarcopenia is most common. In this pilot study, our aim was to determine if mitochondrial respiratory chain function and content are preserved among healthy 85-year-olds.

**Methods:**

We recruited 19 participants (11 female) through their general practitioner and assessed their medical history, functional status and self-reported physical activity. We identified sarcopenia using grip strength, Timed Up-and-Go and bioimpedance analysis. We assessed mitochondrial respiratory chain function using phosphorous magnetic resonance spectroscopy, estimating τ_1/2_ PCr, the recovery half-time of phosphocreatine in the calf muscles following a bout of aerobic exercise. We performed a biopsy of the vastus lateralis muscle and assessed mitochondrial respiratory chain content by measuring levels of subunits of complex I and IV of the respiratory chain, expressed as *Z*-scores relative to that in young controls.

**Results:**

Participants had a median (IQR) of 2 (1,3) long-term conditions, reported regular aerobic physical activity, and one participant (5.3%) had sarcopenia. Sixteen participants completed the magnetic resonance protocol and the mean (SD) τ_1/2_ PCr of 35.6 (11.3) seconds was in keeping with preserved mitochondrial function. Seven participants underwent muscle biopsy and the mean fibre *Z*-scores were −0.7 (0.7) and −0.2 (0.4) for complexes I and IV, respectively, suggesting preserved content of mitochondrial respiratory chain enzymes.

**Conclusion:**

Muscle mitochondrial respiratory chain function and content are preserved in a sample of active, well-functioning 85-year-olds, among whom sarcopenia was uncommon. The results from this study will help inform future work examining the association between muscle mitochondrial deficiency and sarcopenia.

## 1 Introduction

Impairments in skeletal muscle mitochondrial function and content have been implicated in the development of sarcopenia, the age-related loss of muscle mass and performance (Brierley et al., 1996; St-Jean-Pelletier et al., 2017; Joseph et al., 2012). The assessment of skeletal muscle mitochondria presents challenges. Assessment of content, such as staining for cytochrome oxidase, requires the collection of muscle tissue, as do *in vitro* measures of function such as respirometry of isolated mitochondria (Hepple, 2014). *In vivo* measurement of function is possible using phosphorous magnetic resonance spectroscopy (^31^P-MRS), requiring participants to undertake controlled exercise sufficient to deplete muscle reserves of phosphocreatine (Hollingsworth et al., 2008).

As expected for an age-related condition, sarcopenia is most common among the very old (Cruz-Jentoft et al., 2014), with a prevalence of 21% in a sample of 85-year-olds (Dodds et al., 2017). There have been few studies of mitochondrial function and content in this age group, with relevant studies typically having a mean age below 85 (Coen et al., 2013; Choi et al., 2016; Spendiff et al., 2016; Rygiel et al., 2017; Distefano et al., 2017). The opportunity to collect muscle samples during hip fracture surgery has been used to investigate whether impaired mitochondrial homeostasis is associated with sarcopenia among the very old (Marzetti et al., 2016). Older patients with hip fracture are recognised to have not only high levels of sarcopenia but also disability and multimorbidity (Di Monaco et al., 2011; Krishnan et al., 2014); in this setting, the influences of ageing *per se* and those of acute illness and overall frailty may be difficult to disentangle.

A complementary approach is to study community-dwelling very old individuals, including those with few medical and functional problems, who may provide important insights into factors that promote healthy ageing (Ferrucci, 2008). We therefore undertook a pilot study in which we assessed the feasibility of recruiting community-dwelling 85-year-old people to attend for detailed phenotyping including ^31^P-MRS and muscle biopsy. The aim of the present study was to determine if skeletal muscle mitochondrial respiratory chain function and content are preserved among healthy 85-year-olds.

## 2 Methods

### 2.1 Participants

We recruited participants aged 85 years, born in 1931, who were registered with a general practice within the North East & North Cumbria Clinical Research Network, England. We excluded those with a cardiac pacemaker or any other metallic or programmable device (*e*.*g*. cochlear implants or surgical clips) or those who were taking anticoagulant drugs. We also excluded individuals considered unsuitable for approach by their general practitioner (GP). All participants needed to have capacity to provide written informed consent.

Potential participants were identified through their GPs and were sent a letter of invitation, a study information pack and a letter of support from their GP. Individuals expressing an interest in the study were then contacted by the research team and an appointment made to visit them in their own home. At this visit, the requirements of the study were discussed in detail and initial informed consent obtained. Endurance of consent was verified at each contact and prior to any research procedure throughout the research process. The study was approved in the UK by the Tyne & Wear South Research Ethics Committee (15/NE/0382). Fieldwork took place between May and August 2016.

We asked participants whether they had ever been diagnosed by a doctor with 11 common conditions (heart attack, congestive heart failure, angina, stroke/mini-stroke/TIA, hypertension, diabetes, asthma, depression, chronic lung disease, kidney disease or cancer) and recorded their regular prescribed medications. We used the 15-item geriatric depression scale (GDS) and the mini-mental state examination (MMSE) to assess mood and cognition, respectively. We enquired about difficulty or needing help across 17 activities of daily living such as dressing/undressing, cutting toenails, shopping and managing finances. We used the Short Form 36 (SF-36) Health Survey Questionnaire to derive general health and physical functioning scores (Ware and Gandek, 1998). We assessed physical activity using the rapid assessment of physical activity (RAPA), deriving scores for aerobic activity (1–7, with 7 being most active) and strength and flexibility activity (0–3, with 3 most being active) (Topolski et al., 2006).

### 2.2 Identification of sarcopenia

We measured grip strength (kg) with a Jamar handheld hydraulic dynamometer (Promedics, UK) using three trials in both hands following a standard protocol (Roberts et al., 2011) and using the maximum value obtained for analyses. Participants completed the Timed Up-and-Go (TUG) test: a stopwatch was used to measure the time taken to get up from a chair and walk as quickly and safely as possible up to and around a marker placed 3 m away, walk back to the chair and sit back down. We converted this time to an estimate of gait speed (m/s) using the formula (6 / [TUG time]) ∗ 1.62 (Cooper et al., 2011; Cooper et al., 2015). We measured total body weight (kg) and estimated appendicular lean mass (kg) using a Tanita MC-780MA body composition analyser (Tanita Corporation, Arlington Heights, IL.). We estimated height based on demi-span, measured twice to the nearest millimetre. We calculated skeletal muscle index (SMI) (kg/m^2^) from appendicular lean mass divided by height-squared. We applied the European Working Group sarcopenia definition to our results, using recognised cut-points for grip strength of <30 kg in men and <20 kg in women, for gait speed of ≤0.8 m/s and for SMI of <7.26 kg/m^2^ in men and <5.45 kg/m^2^ in women (Cruz-Jentoft et al., 2010). We considered participants with weak grip and/or slow gait speed, in combination with low SMI, to have sarcopenia.

### 2.3 Phosphorous magnetic resonance spectroscopy

**Fig. 1:**
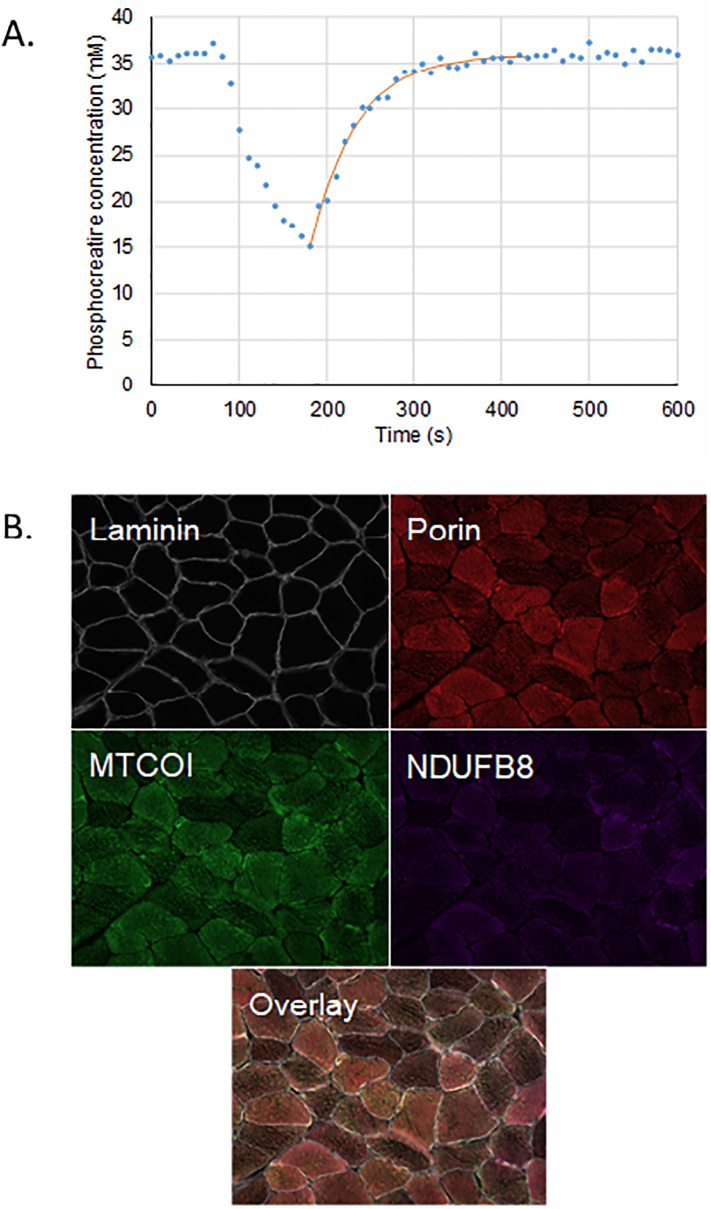
Assessment of mitochondrial function and content. Sample results for mitochondrial function and content (NB two different participants are shown). A. Exponential recovery curve for phosphocreatine following exercise. τ_1/2_ PCr for participant shown is 36.6 s. B. Quadruple immunofluorescence. Mean Z-scores from participant shown of 0.6 for complex IV (MTCOI) and 0.1 for complex I (NDUFB8).

Participants attended for ^31^P-MRS scanning and were requested to perform a low-intensity plantar flexion exercise in the scanner with incremental loading, until the phosphocreatine in the gastrocnemius and soleus muscles was depleted by approximately 50%. Measurements were taken every 10 s during exercise and recovery. We fitted an exponential recovery curve to the area under the phosphocreatine peak from which we modelled the time taken, τ_1/2_ PCr (seconds), for recovery halfway to baseline, as a measure of mitochondrial oxidative function, with shorter times implying higher function (Hollingsworth et al., 2008) (Fig. 1).

### 2.4 Muscle biopsy

We obtained biopsy of the vastus lateralis muscle under local anaesthesia from seven participants using a Weil Blakesley conchotome. The samples were snap frozen in isopentane cooled in liquid nitrogen. We telephone participants the following day to check their wellbeing and visited them at home one week after their biopsy to check the wound had healed and that there were no signs of infection present. We also enquired about any pain at the site, rated on a scale of 0 (no pain) – 10 (worst pain).

### 2.5 Quadruple immunofluorescence

Two 10 μm sections from each biopsy were used for the quadruple immunofluorescence with antibodies to laminin, NDUFB8 (subunit of complex I), MTCOI (subunit of complex IV) and porin, as described previously (Rygiel et al., 2017; Rocha et al., 2015) (Fig. 1). Control samples were biopsies obtained from five younger patients undergoing orthopaedic surgery (see Supplementary Table 1 for full details). The control and participant sections were reacted the same day with the same batch of antibody and identical concentrations. All exposure times were set and maintained throughout the imaging.

The immunofluorescence data from the fibres in the control samples were used to produce linear regression models for the relationships between levels of complex I and porin, and between complex IV and porin. The regression findings were then used to predict the expected levels of complex I and IV per fibre among study participants based on the fibres' measured porin levels. The measured values in complex I and IV were then expressed as *Z*-scores (the number of standard deviations the measured values were above that predicted by the linear regression models). We classified fibres with Z ≥ −3 (so measured values no lower than 3 standard deviations below that predicted from the relationships seen in young controls) as positive.

### 2.6 Statistical analyses

We calculated descriptive statistics for the variables of interest and tested for differences between participants who did and did not undergo muscle biopsy using the Wilcoxon Rank-Sum test. We performed all analyses using Stata version 14.0 (StataCorp, 2015).

## 3 Results

Table 1 shows the characteristics of the 19 participants recruited to the study. They had a median of two diseases and high levels of self-reported physical function and general health, especially among the women in the sample and when compared to normative data for the same age group (Bowling et al., 1999). They regularly engaged in aerobic physical activity with a mean RAPA score of 4.8 (1.4). Their mean results for the components of sarcopenia were either above the relevant cut-points (gait speed, SMI) or just below (grip strength); as such only one participant had sarcopenia according to the EWGSOP definition.

**Table 1:** Sample characteristics.

Characteristic Mean (SD) unless shown otherwise	Men (*n* = 8)	Women (*n* = 11)	All (*n* = 19)
Age at interview (years)	84.9 (0.3)	85.0 (0.3)	85.0 (0.3)
Disease count [median (IQR)]	3 (2,3)	1 (1,3)	2 (1, 3)
No. of prescribed medications [median (IQR)]	7 (3,14)	5 (4,6)	6 (3, 11)
Geriatric depression score	2 (1,2)	1 (0,2)	1 (0, 2)
Mini-mental state examination score	29 (29,30)	30 (29,30)	29 (29, 30)
Number of ADLs with difficulty/help needed	2 (0,3)	0 (0,2)	1 (0, 2)
SF-36 self-reported physical function (0−100)^a^	64.2 (23.4)	79.4 (16.5)	73.1 (20.6)
SF-36 self-reported general health (0–100)^a^	70 (9.6)	79.6 (10.1)	75.5 (10.8)
RAPA aerobic activity score (1–7)^a^	4.6 (1.6)	5 (1.3)	4.8 (1.4)
RAPA strength and flexibility score (0–3)^a^	0.9 (1.0)	0.6 (0.8)	0.7 (0.9)
Grip strength (kg)	29.9 (6.2)	19.1 (6.1)	N/A
Gait speed (m/s)	0.9 (0.4)	1.1 (0.3)	1.0 (0.3)
BMI (kg/m^2^)	27.6 (3.1)	24.4 (4.2)	25.7 (4.0)
SMI (kg/m^2^)	8.1 (0.8)	6.4 (1.0)	N/A
EWGSOP sarcopenia [n (%)]	1 (12.5%)	0 (0%)	1 (5.3%)

ADLs, activities of daily living. BMI, body mass index. EWGSOP, European Working Group on Sarcopenia in Older People. IQR, interquartile range. RAPA, rapid assessment of physical activity. SF-36, Short Form 36 Health Survey Questionnaire. SMI, skeletal muscle index.

a Higher values on the SF-36 and RAPA scores indicate higher levels of function/health and activity, respectively.

### 3.1 Mitochondrial function assessed using magnetic resonance spectroscopy

We collected valid ^31^P-MRS data in 16 participants (one was unable to attend, one declined the scan and in one participant their phosphocreatine did not deplete adequately during exercise). The mean τ_1/2_ PCr was 35.6 (11.3) seconds, in keeping with preserved mitochondrial oxidative capacity (Hollingsworth et al., 2008). The scan procedure was well tolerated by all participants who undertook the test.

### 3.2 Mitochondrial content assessed using quadruple immunofluorescence

Seven participants had a muscle biopsy collected. Reasons for non-participation included the presence of visible veins over the planned biopsy site (*n* = 5), use of medications that could increase risk of bleeding or poor wound-healing (*n* = 3) and participants being unavailable (*n* = 1) or unwilling (n = 3) to have biopsy. We saw no differences in the baseline characteristics (as shown in Table 1) between those participants who did and those who did not have muscle biopsy. There was little evidence of deficiency in men or women of either of the two mitochondrial respiratory chain complexes tested, as shown in Table 2. There were no complications noted at the follow-up home visit and none of the seven participants reported any pain at the biopsy site.

**Table 2:** Mitochondrial function and content.

Characteristic Mean (SD)	Men	Women	All
Phosphorous magnetic resonance spectroscopy	*n* = 6	*n* = 10	*n* = 16
Phosphocreatine recovery rate, τ_1/2_ PCr (s)	33.1 (8.6)	37.0 (12.9)	35.6 (11.3)
Quadruple immunofluorescence	n = 3	*n* = 4	*n* = 7
Complex I			
Mean fibre *Z*-score	−0.5 (1.1)	−0.7 (0.4)	−0.7 (0.7)
Proportion of positive fibres (Z ≥ −3) (%)	98.9 (1.4)	98.6 (0.2)	98.7 (0.9)
Complex IV			
Mean fibre Z-score	0.0 (0.4)	−0.3 (0.5)	−0.2 (0.4)
Proportion of positive fibres (Z ≥ −3) (%)	99.4 (0.4)	98.8 (0.3)	99.1 (0.5)

## 4 Discussion

In this pilot study we carried out an initial investigation of skeletal muscle mitochondrial respiratory chain function and content in an active and healthy sample of 85-year-olds, among whom sarcopenia was uncommon. We found that phosphocreatine recovery time from ^31^P-MRS (in 17 participants) and levels of subunits of complexes I and IV from quadruple immunofluorescence (in 7 participants) were preserved. Both assessments were well tolerated.

Reduction in content and changes to the functions of skeletal muscle mitochondria including reduced respiratory chain function, sensitisation to permeability transition and impaired quality control may contribute to the development of sarcopenia (Hepple, 2014; Marzetti et al., 2013). In addition to those used in the present study, a range of techniques have been used to investigate these changes. For content these include histochemistry of enzymes such as cytochrome *c* oxidase (Brierley et al., 1996) and mtDNA copy number (Short et al., 2005). For functions they include *ex*-*vivo* measurement of respiration in permeabilized myofibres (Spendiff et al., 2016; Distefano et al., 2017) and Western immunoblotting of proteins regulating quality control processes (Marzetti et al., 2016).

There is debate on the extent to which age-related changes in mitochondria represent a primary organelle defect or occur secondary to concomitant reductions in physical activity and cardiorespiratory fitness. Several studies have compared the mitochondrial function and content in young active individuals to that seen in older active and older sedentary groups (St-Jean-Pelletier et al., 2017; Spendiff et al., 2016; Safdar et al., 2010; Distefano et al., 2018) and to that seen in sedentary individuals across a range of ages (Distefano et al., 2017). They have shown that increased habitual physical activity appears to attenuate age-related declines in mitochondrial function and content, with evidence from exercise intervention studies supporting this (Broskey et al., 2014; Lundby and Jacobs, 2016). Therefore, the regular aerobic activity undertaken by participants in our study may have contributed to the preserved mitochondrial function and content.

We are not aware of other data for ^31^P-MRS of the gastrocnemius and soleus muscles in the very old. Taylor et al. previously reported a similar mean τ_1/2_ PCr of 32 s in these muscles following exercise in a sample of six healthy men and women aged 70–83 years (Taylor et al., 1997). Two previous studies have reported ^31^P-MRS data for the quadriceps from three samples with mean ages between 72 and 79 years (Santanasto et al., 2016; Zane et al., 2017), expressed as the recovery rate constant, k_PCr_. If we assume that the two muscle sites are comparable and convert k_PCr_ to τ_1/2_ PCr (using the formula τ_1/2_ PCr = −ln(0.5) / k_PCr_), then their summary values are again similar to our own: ranging from 33.0 to 36.5 s. It is likely that we do not see abnormally long values in our older sample as they regularly engage in aerobic physical activity. This has been shown to have marked benefits for function assessed by ^31^P-MRS of the quadriceps in a sample of young men (Yoshida, 2002), and more recently when comparing older active and sedentary individuals at mean ages 68 and 71, respectively (Distefano et al., 2018).

We also saw largely positive fibres on quadruple immunofluorescence, with mean *Z*-scores of the complex I and IV subunits in the positive range (Z ≥ −3), as we previously reported in a sample of community-dwelling older men at mean age 73 (Rygiel et al., 2017). The high level of physical activity undertaken by our participants may have attenuated the age-related decline in mitochondrial respiratory enzyme content (St-Jean-Pelletier et al., 2017; Distefano et al., 2018; Brierley et al., 1997).

We found that it was feasible to undertake ^31^P-MRS including exercise of the calf muscles in a healthy sample of 85-year-olds and that the procedure was well tolerated. The majority of participants were also willing to undergo biopsy and those who did reported little discomfort following the procedure, in keeping with existing research (Patel et al., 2011; Baczynska et al., 2017). We exercised caution when deciding to proceed with biopsy, for example excluding participants with visible veins around the biopsy site. Understanding more about the acceptability and feasibility of muscle biopsy in the very old would allow cellular and molecular mechanistic studies in this age group to flourish.

This study had several strengths. We successfully recruited a sample of healthy 85-year-old people for detailed phenotyping related to skeletal muscle. We carried out an initial home visit, giving participants opportunity to meet a member of the study team and discuss what the study involved; this has previously been linked to engagement with intensive assessments such as muscle biopsy (Baczynska et al., 2017).

Limitations of this study include the fact that we assessed physical activity using a questionnaire; an objective measurement would have given us useful additional information regarding the intensity and patterns of activity. We did not attempt muscle biopsy in around two-thirds of participants, mainly due to skin changes or medication history. Our sample was also biased towards healthier and more active participants, among whom sarcopenia and other conditions were less common than average for this age group (Dodds et al., 2017; Collerton et al., 2016). This may in part reflect the study's exclusion criteria: for example, those taking anticoagulant drugs are more likely to have cardiovascular disease than the general population. The small sample size of our study prevented us from examining associations between mitochondrial function/content and the components of sarcopenia. The small sample sizes and variability of the measures we report also suggest that the mean values we show in Table 2 may well not be representative of the underlying population.

In conclusion, we found that skeletal muscle respiratory chain function, assessed using ^31^P-MRS, and the content of two respiratory chain subunits in muscle biopsy samples were preserved in a healthy, active sample of 85-year-old men and women. This is likely to reflect the fact that our sample reported regularly engaging in aerobic exercise. These results will help to inform future studies in this age group, including in those with lower activity levels and higher levels of sarcopenia than in this pilot study.

Declarations

## Funding

This study was funded by a Medical Research Council Confidence in Concept grant to AAS.

The research was supported by the NIHR Newcastle Biomedical Research Centre awarded to the Newcastle upon Tyne Hospitals NHS Foundation Trust and Newcastle University, Wellcome Centre for Mitochondrial Research (203105/Z/16/Z), the Medical Research Council (MRC) Centre for Translational Research in Neuromuscular Disease, Newcastle University Centre for Ageing and Vitality (supported by the Biotechnology and Biological Sciences Research Council and MRC) and Mitochondrial Disease Patient Cohort (UK) (G0800674). The views expressed are those of the author(s) and not necessarily those of the NHS, the NIHR or the Department of Health.

The following is the supplementary data related to this article.

Supplementary Table 1 Details of controls.
Five younger patients gave written consent for muscle biopsy at the time of orthopaedic surgery. Supplementary Table 1

## Conflicts of interest

The authors have no conflicts of interest to declare.

## References

[bb0005] Baczynska A.M., Shaw S.C., Patel H.P., Sayer A.A., Roberts H.C. (2017). Learning from older peoples' reasons for participating in demanding, intensive epidemiological studies: a qualitative study. BMC Med. Res. Methodol..

[bb0010] Bowling A., Bond M., Jenkinson C., Lamping D.L. (1999). Short form 36 (SF-36) health survey questionnaire: which normative data should be used? Comparisons between the norms provided by the omnibus survey in Britain, the health survey for England and the Oxford healthy life survey. J. Public Health Med..

[bb0015] Brierley E.J., Johnson M.A., OF James, Turnbull D.M. (1996). Effects of physical activity and age on mitochondrial function. QJM.

[bb0020] Brierley E.J., Johnson M.A., Bowman A. (1997). Mitochondrial function in muscle from elderly athletes. Ann. Neurol..

[bb0025] Broskey N.T., Greggio C., Boss A. (2014). Skeletal muscle mitochondria in the elderly: effects of physical fitness and exercise training. J. Clin. Endocrinol. Metab..

[bb0030] Choi S., Reiter D.A., Shardell M. (2016). 31P magnetic resonance spectroscopy assessment of muscle bioenergetics as a predictor of gait speed in the Baltimore longitudinal study of aging. J. Gerontol. A Biol. Sci. Med. Sci..

[bb0035] Coen P.M., Jubrias S.A., Distefano G. (2013). Skeletal muscle mitochondrial energetics are associated with maximal aerobic capacity and walking speed in older adults. J. Gerontol. A Biol. Sci. Med. Sci..

[bb0040] Collerton J., Jagger C., Yadegarfar M.E. (2016). Deconstructing complex multimorbidity in the very old: findings from the Newcastle 85+ study. Biomed. Res. Int..

[bb0045] Cooper R., Hardy R., Aihie Sayer A. (2011). Age and gender differences in physical capability levels from mid-life onwards: the harmonisation and meta-analysis of data from eight UK cohort studies. PLoS One.

[bb0050] Cooper R., Bann D., Wloch E.G., Adams J.E., Kuh D. (2015). “Skeletal muscle function deficit” in a nationally representative British birth cohort in early old age. J. Gerontol. A Biol. Sci. Med. Sci..

[bb0055] Cruz-Jentoft A.J., Baeyens J.P., Bauer J.M. (2010). Sarcopenia: European consensus on definition and diagnosis: report of the European Working Group on sarcopenia in older people. Age Ageing.

[bb0060] Cruz-Jentoft A.J., Landi F., Schneider S.M. (2014). Prevalence of and interventions for sarcopenia in ageing adults: a systematic review. Report of the International Sarcopenia Initiative (EWGSOP and IWGS). Age Ageing.

[bb0065] Di Monaco M., Vallero F., Di Monaco R., Tappero R. (2011). Prevalence of sarcopenia and its association with osteoporosis in 313 older women following a hip fracture. Arch. Gerontol. Geriatr..

[bb0070] Distefano G., Standley R.A., Dubé J.J. (2017). Chronological age does not influence ex-vivo mitochondrial respiration and quality control in skeletal muscle. J. Gerontol. A Biol. Sci. Med. Sci..

[bb0075] Distefano G., Standley R.A., Zhang X. (2018). Physical activity unveils the relationship between mitochondrial energetics, muscle quality, and physical function in older adults. J. Cachexia. Sarcopenia Muscle.

[bb0080] Dodds R.M., Granic A., Davies K., Kirkwood T.B.L., Jagger C., Sayer A.A. (2017). Prevalence and incidence of sarcopenia in the very old: findings from the Newcastle 85+ study. J. Cachexia. Sarcopenia Muscle.

[bb0085] Ferrucci L. (2008). The Baltimore longitudinal study of aging (BLSA): a 50-year-long journey and plans for the future. J. Gerontol. A Biol. Sci. Med. Sci..

[bb0090] Hepple R.T. (2014). Mitochondrial involvement and impact in aging skeletal muscle. Front. Aging Neurosci..

[bb0095] Hollingsworth K.G., Newton J.L., Taylor R. (2008). Pilot study of peripheral muscle function in primary biliary cirrhosis: potential implications for fatigue pathogenesis. Clin. Gastroenterol. Hepatol..

[bb0100] Joseph A.M., Adhihetty P.J., Buford T.W. (2012). The impact of aging on mitochondrial function and biogenesis pathways in skeletal muscle of sedentary high- and low-functioning elderly individuals. Aging Cell.

[bb0105] Krishnan M., Beck S., Havelock W., Eeles E., Hubbard R.E., Johansen A. (2014). Predicting outcome after hip fracture: using a frailty index to integrate comprehensive geriatric assessment results. Age Ageing.

[bb0110] Lundby C., Jacobs R.A. (2016). Adaptations of skeletal muscle mitochondria to exercise training. Exp. Physiol..

[bb0115] Marzetti E., Calvani R., Cesari M. (2013). Mitochondrial dysfunction and sarcopenia of aging: from signaling pathways to clinical trials. Int. J. Biochem. Cell Biol..

[bb0120] Marzetti E., Calvani R., Lorenzi M. (2016). Association between myocyte quality control signaling and sarcopenia in old hip-fractured patients: results from the sarcopenia in HIp FracTure (SHIFT) exploratory study. Exp. Gerontol..

[bb0125] Patel H., Syddall H.E., Martin H.J., Cooper C., Stewart C., Sayer A.A. (2011). The feasibility and acceptability of muscle biopsy in epidemiological studies: findings from the Hertfordshire Sarcopenia Study (HSS). J. Nutr. Health Aging.

[bb0130] Roberts H.C., Denison H.J., Martin H.J. (2011). A review of the measurement of grip strength in clinical and epidemiological studies: towards a standardised approach. Age Ageing.

[bb0135] Rocha M.C., Grady J.P., Grünewald A. (2015). A novel immunofluorescent assay to investigate oxidative phosphorylation deficiency in mitochondrial myopathy: understanding mechanisms and improving diagnosis. Sci. Rep..

[bb0140] Rygiel K.A., Dodds R.M., Patel H.P. (2017). Mitochondrial respiratory chain deficiency in older men and its relationship with muscle mass and physical performance: findings from the Hertfordshire Sarcopenia Study. J. Cachexia. Sarcopenia Muscle.

[bb0145] Safdar A., Hamadeh M.J., Kaczor J.J., Raha S., de Beer J., Tarnopolsky M.A. (2010). Aberrant mitochondrial homeostasis in the skeletal muscle of sedentary older adults. PLoS One.

[bb0150] Santanasto A.J., Coen P.M., Glynn N.W. (2016). The relationship between mitochondrial function and walking performance in older adults with a wide range of physical function. Exp. Gerontol..

[bb0155] Short K.R., Bigelow M.L., Kahl J. (2005). Decline in skeletal muscle mitochondrial function with aging in humans. Proc. Natl. Acad. Sci. U. S. A..

[bb0160] Spendiff S., Vuda M., Gouspillou G. (2016). Denervation drives mitochondrial dysfunction in skeletal muscle of octogenarians. J. Physiol..

[bb0165] StataCorp (2015). Stata Statistical Software: reLease 14.

[bb0170] St-Jean-Pelletier F., Pion C.H., Leduc-Gaudet J.-P. (2017). The impact of ageing, physical activity, and pre-frailty on skeletal muscle phenotype, mitochondrial content, and intramyocellular lipids in men. J. Cachexia. Sarcopenia Muscle.

[bb0175] Taylor D.J., Kemp G.J., Thompson C.H., Radda G.K. (1997). Ageing: effects on oxidative function of skeletal muscle in vivo. Mol. Cell. Biochem..

[bb0180] Topolski T.D., Logerfo J., Patrick D.L., Williams B., Walwick J., Patrick M.B. (2006). The Rapid Assessment of Physical Activity (RAPA) among older adults. Prev. Chronic Dis..

[bb0185] Ware J.E., Gandek B. (1998). Overview of the SF-36 health survey and the International Quality of Life Assessment (IQOLA) project. J. Clin. Epidemiol..

[bb0190] Yoshida T. (2002). The rate of phosphocreatine hydrolysis and resynthesis in exercising muscle in humans using 31P-MRS. J. Physiol. Anthropol. Appl. Hum. Sci..

[bb0195] Zane A.C., Reiter D.A., Shardell M. (2017). Muscle strength mediates the relationship between mitochondrial energetics and walking performance. Aging Cell.

