# Supplementary material for: Mitochondrial respiratory chain function and content are preserved in the skeletal muscle of active very old men and women
Source: Exp Gerontol. 2018 Nov;113:80–5. doi: 10.1016/j.exger.2018.09.020 (PMC6224654; doi:10.1016/j.exger.2018.09.020)
Supplement: Supplementary Table 1 — Details of controls. Five younger patients gave written consent for muscle biopsy at the time of orthopaedic surgery. [file mmc1.docx]

Supplementary Table 1

*Details of controls*

Five younger patients gave written consent for muscle biopsy at the time of orthopaedic surgery.

| Control | Gender | Age (years) |
| --- | --- | --- |
| 1 | Male | 17 |
| 2 | Male | 33 |
| 3 | Male | 28 |
| 4 | Female | 25 |
| 5 | Female | 23 |
